# Ceruloplasmin oxidized and deamidated by Parkinson's disease cerebrospinal fluid induces epithelial cells proliferation arrest and apoptosis

**DOI:** 10.1038/s41598-020-72447-z

**Published:** 2020-09-23

**Authors:** Marco Barbariga, Alan Zanardi, Flavio Curnis, Antonio Conti, Daniela Boselli, Simona Di Terlizzi, Massimo Alessio

**Affiliations:** 1 Proteome Biochemistry, IRCCS-Ospedale San Raffaele, via Olgettina 58, 20132 Milan, Italy; 2 Tumor Biology and Vascular Targeting, IRCCS-Ospedale San Raffaele, 20132 Milan, Italy; 3 FRACTAL (Flow Cytometry Resource, Advanced Cytometry Technical Applications Laboratory), IRCCS-Ospedale San Raffaele, 20132 Milan, Italy

**Keywords:** Neuroscience, Diseases of the nervous system, Parkinson's disease

## Abstract

In Parkinson's disease, the ferroxidase ceruloplasmin (Cp) is oxidized and deamidated by the pathological cerebrospinal fluid (CSF) environment. These modifications promote the gain of integrin binding properties, fostered by the deamidation of two NGR-motifs present in the Cp sequence that convert into the isoDGR-motif. Through isoDGR/integrin binding, the oxidized/deamidated-Cp (Cp-ox/de) mediates cell adhesion and transduces an intracellular signal in epithelial cells that seems to be addressed to regulate cell cycle, proliferation and cytoskeletal re-arrangement. However, the effect fostered on cells by integrins engagement via Cp-ox/de is not known. We found that in HaCaT epithelial cells, the incubation with Cp-ox/de resulted in proliferation inhibition mediated by isoDGR, cell cycle arrest and apoptosis induction. Similar proliferation inhibition was induced by treatment with purified Cp previously incubated in the CSF from Parkinson's disease patients, but not by Cp incubated in the CSF from healthy subjects. In human primary choroid plexus epithelial cells, a possible in vivo target of Cp-ox/de generated in pathological CSFs, we found that Cp-ox/de mediated cell adhesion via isoDGR/integrins binding and transduced an intracellular signal, which resulted in cell proliferation inhibition. Thus, the generation of Cp-ox/de in pathological CSFs and the consequent apoptosis induction of epithelial cells facing the liquor, might represent a novel mechanism that contributes to neurodegeneration.

## Introduction

Alterations in cerebrospinal fluid (CSF) composition are reported in aging and neurodegenerative diseases, such as Parkinson's disease (PD), Alzheimer’s disease (AD), and multiple sclerosis^1,2^. In addition to changes in protein levels, large part of the alterations are promoted by the pro-oxidant pathological environment of the CSF which in turn fosters protein oxidative modifications^3–5^. These modifications have been used for identification of putative biomarkers aimed to help early and/or differential diagnosis^5,6^. Nevertheless, such protein modifications in the CSF may themselves contribute to pathological mechanisms and disease progression.

Ceruloplasmin (Cp) is a ferroxidase enzyme present in the central nervous system (CNS) as soluble isoform in the CSF and as glycosylphosphatidylinositol-membrane anchored isoform in astrocytes. Cp regulates cellular iron loading and export in neuron and glial cells, and hence protects CNS from iron-mediated free radical injury^7–9^. For these reasons, Cp has been proposed to have a role in several neurodegenerative diseases^10–12^. Such role is underlined in aceruloplasminemia, a monogenic rare disease in which the absence of Cp ferroxidase activity results, *inter alia,* in neurodegeneration due to brain iron accumulation^13^, and the Cp replacement therapy is efficacious in preventing neurodegeneration progression^14^. Cp was reported to be oxidized in the CSF of PD and AD patients, likely as consequence of the oxidative pathological environment^5^. Indeed, spiking of purified Cp in the CSF from PD or AD patients resulted in the same Cp modifications^15,16^. Such modifications promote loss of Cp ferroxidase activity, which in turn fosters intracellular iron accumulation^5,15^. In addition to the loss of enzymatic activity, Cp modifications promote de novo gain of integrin binding properties^15,16^. These latest are acquired by the deamidation of the Asn residue of the Asn-Gly-Arg (NGR)-motifs present in the Cp sequence (N^568^ and N^962^) that lead to a transformation of NGR into the isoAsp-Gly-Arg (isoDGR)-motif which binds several integrins via the RGD-binding site of RGD-integrin family^15,17,18^. Through isoDGR/integrin binding, the Cp-ox/de transduces an intracellular signal that, at the molecular level through FAK1, ERK1/2, Akt and MAPK involvement, seems to be aimed to regulate cell cycle, proliferation, and cytoskeletal re-arrangement in epithelial cells^15^.

In the CSF of PD patients, the endogenous Cp has been found deamidated at the ^962^NGR-motif^16^; while, in vitro, the ^962^NGR-motif underwent deamidation reaction exclusively when protein aging occurred under oxidative conditions that affect the Cp-structure and promote the exposure of the ^962^NGR-motif, usually hidden within the protein^15^.

In this study we report that the incubation with Cp-ox/de affects epithelial cells physiology in terms of cell proliferation, cell cycle arrest and apoptosis induction. Indeed, Cp modified by incubation in the CSF from PD patients is able to induce analogous proliferation inhibition. Most importantly, cell proliferation arrest induced by Cp-ox/de can be significantly rescued by protein-l-isoAsp-O-methyltransferase (PIMT) enzyme treatment, an enzyme that converts isoaspartate to aspartate, suggesting a critical role of isoAsp residues, presumably through the interaction of the Cp isoDGR motifs with the integrins expressed on epithelial cells. Proliferation inhibition is similarly induced by Cp-ox/de on specialized epithelial cells of the choroid plexus whose, in the CNS, face the pathological CSF containing the modified Cp.

These results highlight a mechanism that might contribute to alteration of epithelial cells physiology in neurodegenerative disorders characterized by oxidative pathological environment.

## Results

### Oxidized and deamidated Cp induces proliferation arrest of epithelial HaCaT cells

**Figure 1:**
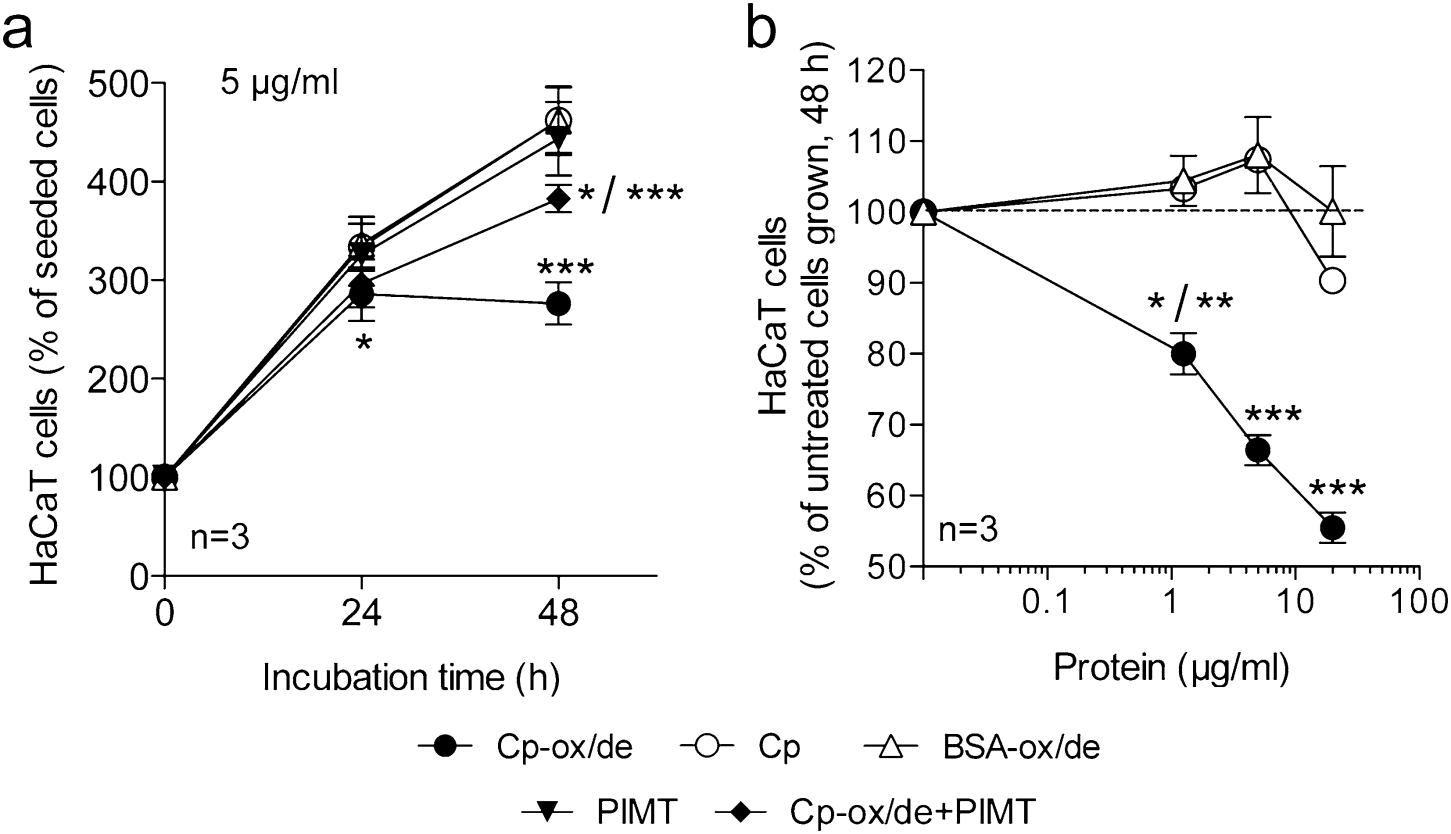
Effect of Cp and oxidized/deamidated-Cp (Cp-ox/de) on the HaCaT epithelial cell proliferation. (**a**) HaCaT cells were incubated for 24 or 48 h with 5 μg/ml of Cp-ox/de, untreated Cp, oxidized/deamidated-BSA (BSA-ox/de), Cp-ox/de treated with PIMT (Cp-ox/de + PIMT) or PIMT alone in the vehicle (PIMT). Cell number was determined by MTT assay and reported as percentages of the seeded cells (see Material and methods). (**b**) HaCaT cells were incubated for 48 h with the indicated amounts of stimuli. Cell proliferation was determined by MTT assay and reported as percentages of the untreated cells grown. Statistical significance p value was evaluated by one way ANOVA (mean ± SE; n = 3, in triplicates) with post analysis Tukey’s test for comparison of all pairs of groups (***p < 0.0001; **p < 0.01; *p < 0.05). (**a**) p < 0.0001, ANOVA; post test at 24 h: *, for Cp-ox/de vs. Cp and Cp-ox/de vs. BSA-ox/de; post test at 48 h: ***, for Cp-ox/de vs. Cp, Cp-ox/de vs. BSA-ox/de, Cp-ox/de vs. PIMT, Cp-ox/de vs. Cp-ox/de + PIMT, Cp vs. Cp-ox/de + PIMT, BSA-ox/de vs. Cp-ox/de + PIMT; *, for for PIMT vs. Cp-ox/de + PIMT. (**b**) p < 0.0001, ANOVA; post test at 1 μg/ml: *, for Cp-ox/de vs. Cp; **, for Cp-ox/de vs. BSA-ox/de; post test at 5 μg/ml and 20 μg/ml: ***, for Cp-ox/de vs. Cp and Cp-ox/de vs. BSA-ox/de.

Since signalling transduction via integrin engagement by Cp-ox/de targets molecules associated with cell cycle and proliferation pathways^15^, we investigated the effects of Cp-ox/de binding to epithelial cells. HaCaT cells treated with Cp-ox/de showed proliferation reduction at 24 h (p < 0.0001, one way ANOVA; Tukey's post test analysis at 24 h: p < 0.05 for Cp-ox/de vs. Cp; Cp-ox/de vs. BSA-ox/de) and proliferation arrest at 48 h (p < 0.0001, one way ANOVA; Tukey's post test analysis at 48 h: p < 0.0001 for Cp-ox/de vs. Cp; Cp-ox/de vs. BSA-ox/de) (Fig. 1a). Proliferation arrest was confirmed by the post test analysis comparison of cell growth from 24 to 48 h of culture under the same experimental conditions. All the conditions showed a significant difference (p < 0.0001), which in turn indicated cell growth, with the exception of Cp-ox/de treatment that resulted not to be significantly different from 24 and 48 h, underlining the block of cell proliferation.

The inhibition was dose-dependent and was not observed when cells were incubated with untreated Cp or with control BSA-ox/de (p < 0.0001, one way ANOVA; Tukey's post test analysis: p < 0.0001 for Cp-ox/de vs. Cp; Cp-ox/de vs. BSA-ox/de at 20 μg/ml and 5 μg/ml; p < 0.05 for Cp-ox/de vs. Cp and p < 0.01 for Cp-ox/de vs. BSA-ox/de at 1 μg/ml) (Fig. 1b). Treatment of Cp-ox/de with PIMT partially, but significantly rescued the cell proliferation (p < 0.0001, one way ANOVA; Tukey's post test analysis at 48 h: p < 0.0001 for Cp-ox/de vs. PIMT; Cp-ox/de vs. Cp-ox/de + PIMT) (Fig. 1a). PIMT is an enzyme that converts L-isoAsp residues to L-Asp, thus converting the isoDGR motifs of the Cp-ox/de to DGR motifs, being the latter not able to bind integrins^15,19^. These results indicated that isoAsp is critically involved in the induction of proliferation inhibition by Cp-ox/de, and suggested that Cp-ox/de might interact with the integrins expressed on the surface of HaCaT cells through its isoDGR-motifs.

The non-complete rescue of cell proliferation by PIMT treatment of Cp-ox/de, was probably due to a not fully enzymatic conversion from isoaspartate to aspartate, that resulted in a residual inhibitory effect on proliferation when compared to controls (p < 0.0001, one way ANOVA; Tukey's post test analysis at 48 h: p < 0.0001 for Cp vs. Cp-ox/de + PIMT; BSA-ox/de vs. Cp-ox/de + PIMT; and p < 0.05 for PIMT vs. Cp-ox/de + PIMT) (Fig. 1a).

To rule out any sensitivity drawbacks of the proliferation assay used, that was based on the 3-(4,5-dimethylthiazol-2-yl)-2,5-diphenyltetrazolium bromide (MTT) salt reduction, the analysis was also performed using the in vivo time-lapse imaging proliferation measurement. The results obtained were comparable to those obtained with MTT, indicating a specific proliferation inhibition induced by Cp-ox/de (Supplementary Information, Figure SI-1a,b,c).

### Cp-ox/de induction of epithelial cells proliferation arrest is the result of cell cycle blockage with consequent apoptosis triggering

**Figure 2:**
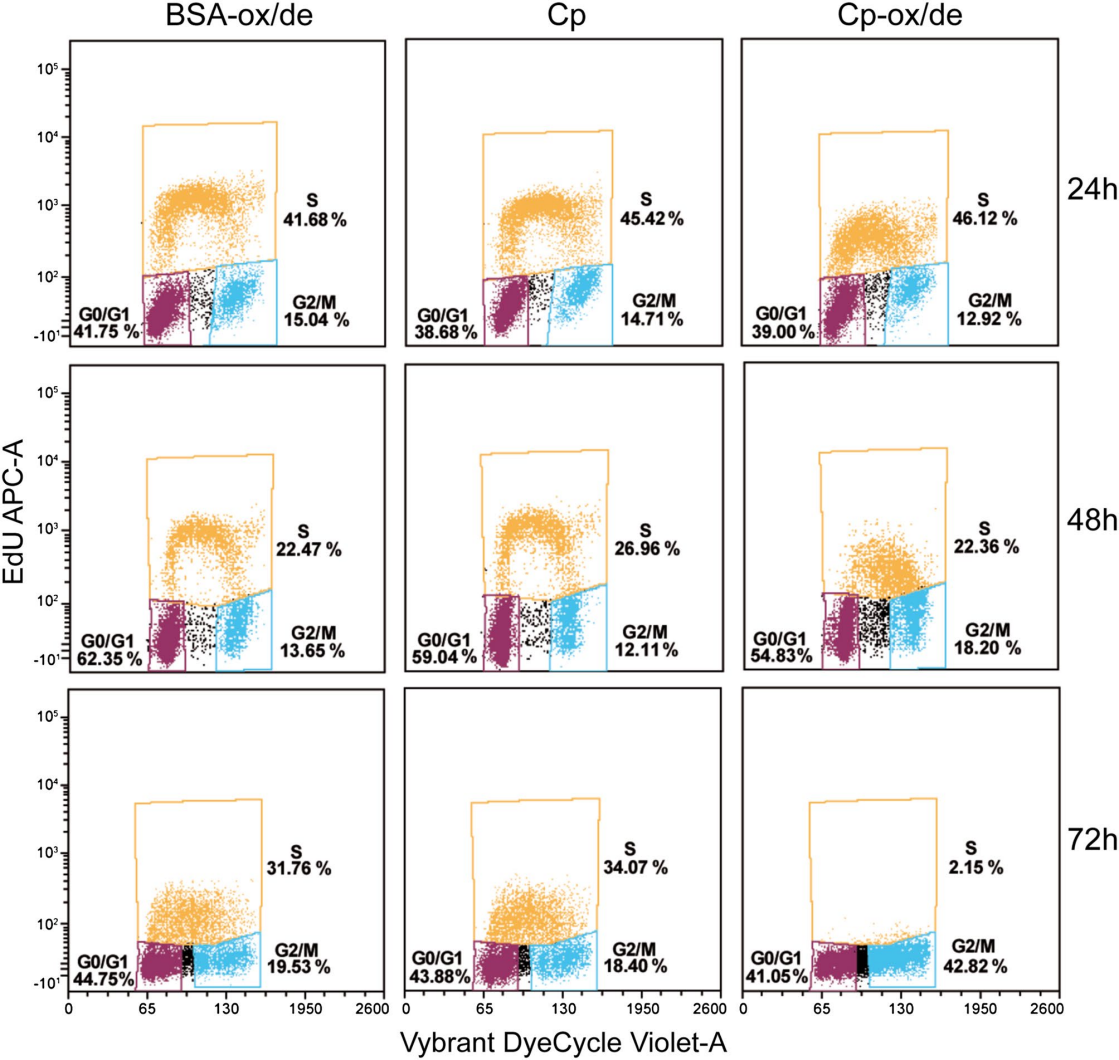
Effect of Cp and Cp-ox/de on HaCaT cell cycle. HaCaT cells were cultured in complete medium containing 0.1% FBS for 24 h and then incubated with Cp, Cp-ox/de or BSA-ox/de (5 μg/ml) for the indicated times. Cells were then stained with a thymidine analog (EdU) in order to discriminate cells in S phase from those in G0/G1 and G2 + M (y axes), and with Vybrant DyeCycle Violet (Invitrogen) for total DNA, discriminating between G0/G1 and G2 phase (x axes) Cells were analyzed by flow cytometry, and percentages of gated cells are reported.

Flow cytometry analysis showed that the proliferation inhibition was the result of cell cycle blockage. In contrast with cells treated either with untreated Cp or with BSA-ox/de, epithelial cells incubated with Cp-ox/de after 48 h started to accumulate in the late-S/G2-entry phase of the cell cycle (Fig. 2); this result suggests that cell arrest occurred in the G0/G1 phase. The effect was more evident at 72 h, when very few cells treated with Cp-ox/de were in the S phase, and accumulation was observable in the G2/M phase (Fig. 2). The rate of EdU incorporation was already reduced in Cp-ox/de treated cells at 24 h, indicating an early effect; analogous reduction was observed for the control treatments (untreated Cp and BSA-ox/de) at 72 h, likely due to the very low serum concentration (0.1%) used in cell culture (Fig. 2).

**Figure 3:**
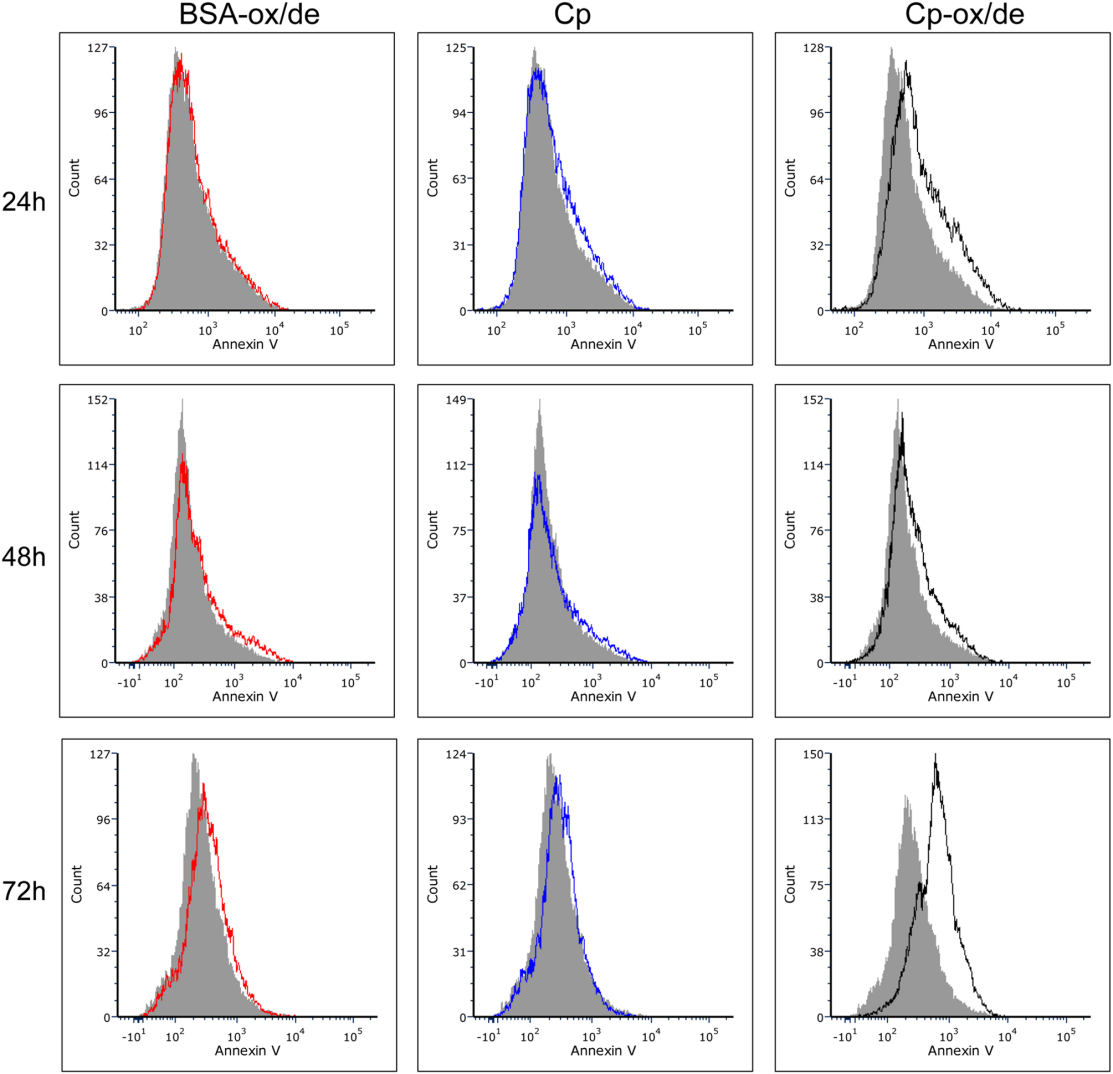
Apoptosis detection by annexin V staining and flow cytometry analysis. HaCaT cells were treated as for cell cycle analysis and stained with annexin V. Percentage of annexin V positive cells is reported for cells after 72 h treatment with Cp-ox/de. The grey profile is referred to control untreated cells. See also Supplementary Information Figure SI-3 for gating strategy used to identify cell singlets.

Annexin V staining revealed that after 72 h of treatment with Cp-ox/de about 48% of the cells were in apoptosis, while almost no annexin V staining was detected in untreated Cp and BSA-ox/de treated cells (Fig. 3).

### Cp modified by incubation in CSF from PD patients reduces HaCaT epithelial cell proliferation

It has been reported that spiked Cp aged ex-vivo in pro-oxidant pathological PD-CSF environment, showed deamidation of the ^962^NGR-motif and the gain of integrin-binding properties^16^. Therefore, to investigate whether Cp aged in pathological CSF environment could also inhibit cell proliferation, we incubated HaCaT cells with Cp immunoprecipitated after spiking and incubation for 0 day or after 9 days of aging in pools of CSF from either healthy subjects (H-CSF) or PD patients (PD-CSF). Pro-oxidant environment of the CSF from PD patients used to generate the CSF pool for Cp spiking and aging, was underlined by their high H_2_O_2_ concentrations, on average 50 μM, compared to concentration found in the CSF of healthy subjects, on average 25 μM^16^.

**Figure 4:**
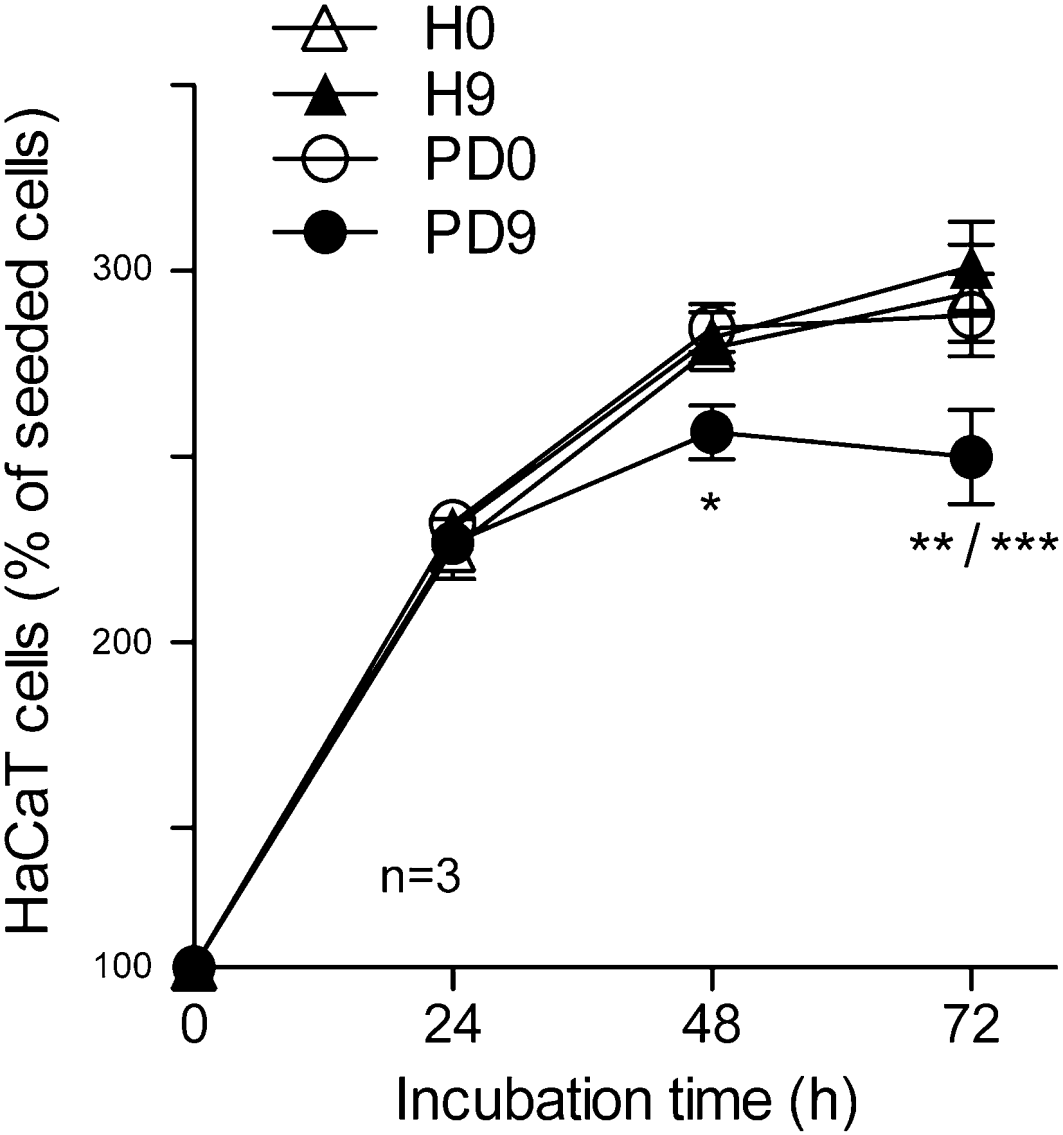
Cp modified by incubation in CSF from PD patients reduces HaCaT epithelial cell proliferation. Proliferation of HaCaT cells was measured by MTT assay upon incubation (24, 48, 72 h) with 2 μg/ml of purified Cp immunoprecipitated after aging (0 or 9 days at 37 °C) in healthy subjects CSF or Parkinson’s disease patients CSF (H-CSF and PD-CSF, respectively). Statistical significance p value was evaluated by one way ANOVA (p < 0.0001) (mean ± SE; n = 3, in triplicates) with post analysis Bonferroni’s test for comparison of selected pairs of groups (*p < 0.05; **p < 0.01; ***p < 0.0001). Post test analysis at 48 h: *, for PD9 vs. PD0 and PD9 vs. H9; post test at 72 h: **, for PD9 *vs.* PD0 and PD9 vs. H0; ***, for PD9 vs. H9.

HaCaT cells treated with Cp derived from aging in PD-CSF showed a reduction in proliferation at 48 h (p < 0.0001, one way ANOVA; Bonferroni's post test analysis: p < 0.05 for PD9 vs. PD0 and PD9 vs. H9) and a proliferation arrest at 72 h (p < 0.0001 one way ANOVA; Bonferroni's post test analysis: p < 0.01 for PD9 vs. PD0 and PD9 vs. H0; p < 0.0001 for PD9 vs. H9) (Fig. 4). Inhibition was absent when Cp aged in healthy CSF (H9) was added to the cell culture, as well as when Cp was not subjected to aging treatment (H0 and PD0, respectively) (Fig. 4). Indeed, the post test analysis comparison of cell growth from 24 to 72 h of culture under the same experimental conditions showed a significant difference (p < 0.0001, Bonferroni's test) for H0, H9 and PD0, which in turn indicated cell growth, while PD9 did not showed significant difference, underlining the block of cell proliferation.

### Cp-ox/de mediates primary human choroid plexus epithelial cells (HCPEpiCs) adhesion via isoDGR/integrins binding

Since in vivo, a possible target of Cp-ox/de generated in the pathological CSF is the choroid plexus whose specialized epithelial cells are in contact with the CSF, we investigated the effects of Cp-ox/de on HCPEpiCs. These cells have been characterized for the expression of integrins reported to be suitable for the binding of the isoDGR motif^15,18^. HCPEpiCs showed surface expression of the αV-subunit of integrins, the αVβ3, and α5β1 heterodimers, but not the αVβ5 and αVβ6 complexes, therefore they can be targeted by Cp-ox/de (Supplementary Information, Figure SI-2).

**Figure 5:**
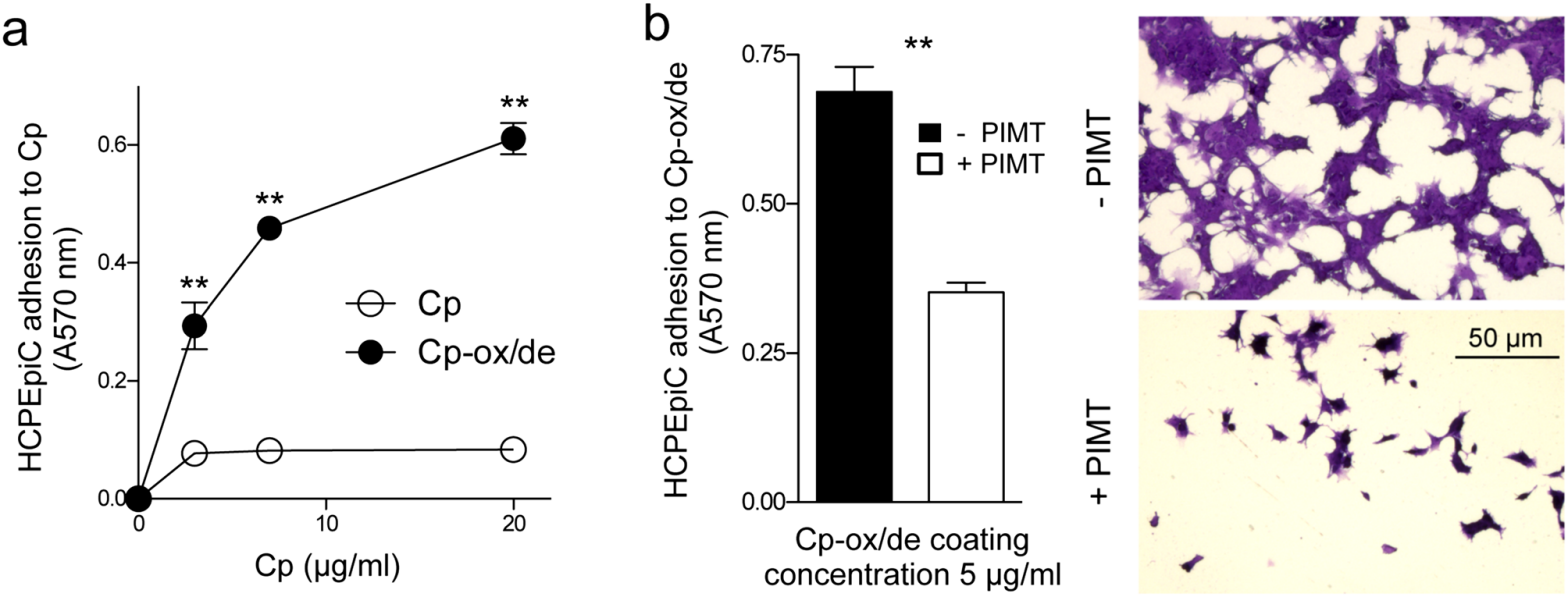
Cp-ox/de mediates HCPEpiCs cell adhesion via isoDGR-integrins interaction. (**a**) Adhesion of HCPEpiCs to plates coated with different concentrations (3, 7, 20 μg/ml) of untreated Cp or Cp-ox/de. Adherents cells were stained with crystal violet and cells adhesion was quantified by measuring the absorbance at 570 nm. Statistical significance p value was evaluated by Kruskal–Wallis test (p = 0.0003; mean ± SE; n = 3, in triplicates) with Dunn’s post test analysis for comparison of selected pairs of groups (**p < 0.01). (**b**) Effect of PIMT enzyme on HCPEpiCs cells adhesion to microtiterwells coated with Cp-ox/de. Quantification of cell adhesion and representative images of HCPEpiCs cells adherent to Cp-ox/de treated with ( +) or without (−) PIMT enzyme are shown. Adhesion assays were performed in 3 independent experiments. Statistical significance p value was evaluated by Mann–Whitney test on mean ± SE of 3 experiment with 3 replicates each; **p = 0.0079.

Similarly to HaCaT epithelial cells^15^, the HCPEpiCs showed a dose-dependent adhesion to plates coated with Cp-ox/de, while coating with untreated Cp was uneffective (Fig. 5a) (p = 0.0003, Kruskal–Wallis test; Dunn's post test Cp vs. Cp-ox/de p < 0.01 at 3, 7 and 20 μg). To confirm that cell adhesion was mediated by the isoDGR motifs present in the Cp-ox/de sequence, we incubated Cp-ox/de with PIMT. Treatment with PIMT enzyme inhibited the pro-adhesive activity of Cp-ox/de (p = 0.0079, Mann–Whitney test) (Fig. 5b), indicating that deamidated Cp interacted with the integrins expressed on the surface of HCPEpiCs cells, through its isoDGR motifs.

### Cp-ox/de mediates HCPEpiCs signal transduction and proliferation inhibition

**Figure 6:**
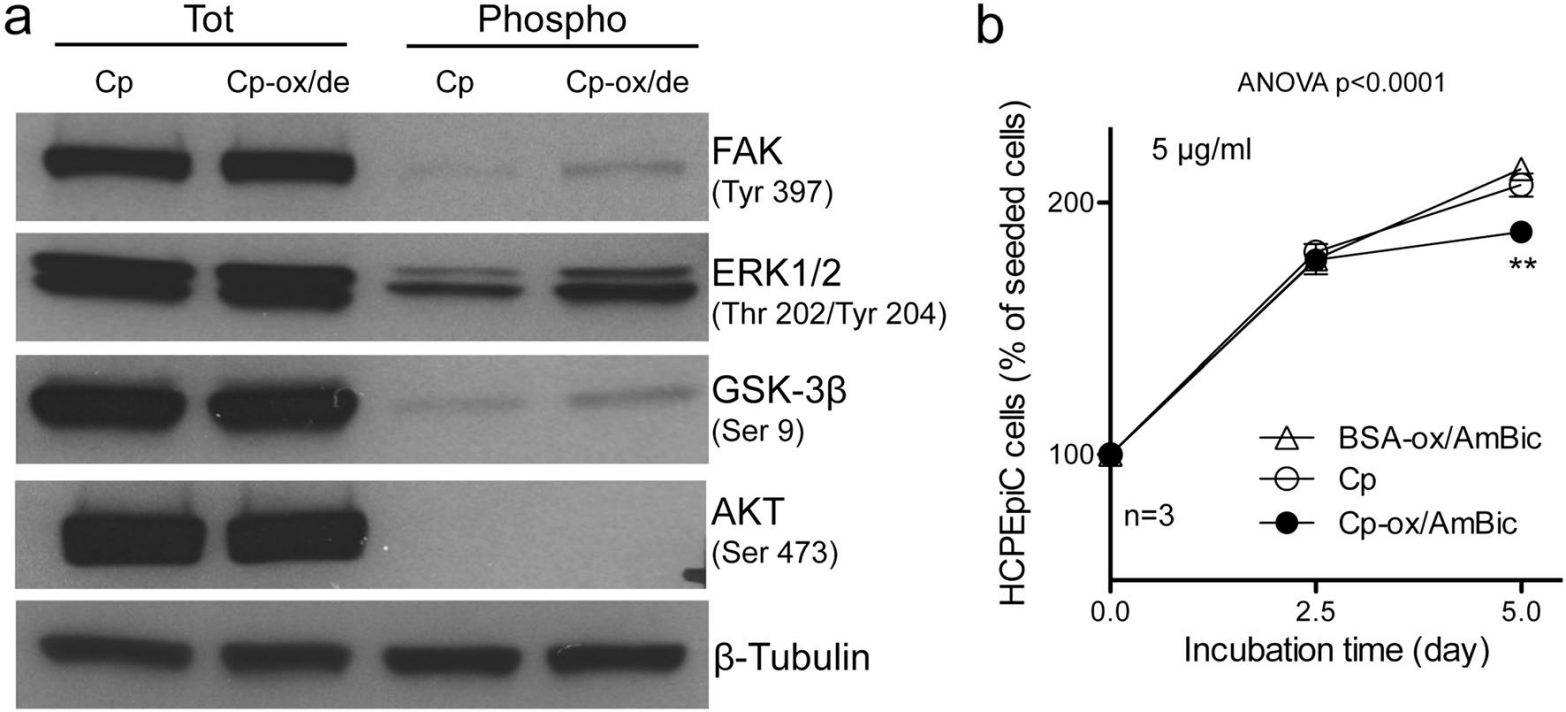
Cp-ox/de transduces an intracellular signalling in HCPEpiC that promotes proliferation inhibition. (**a**) Western blot analysis of the intracellular signalling transduced by Cp-ox/de in HCPEpiCs cells. Cells were treated for 2 h with 5 μg/ml of untreated Cp or Cp-ox/de. Total protein expression (Tot) and phosphorylation of specific residues (Phospho) was evaluated in the cell lysate (FAK and p-Tyr^397^FAK, ERK1/2 and p-Thr^202/^Tyr^204^ERK1/2, GSK3β and p-Ser^9^GSK3β, AKT and p-Ser^473^AKT were evaluated). Gel loading was normalized by β-Tubulin expression. Cropped images of FAK, ERK1/2, AKT and GSK3β are from the same SDS-PAGE gel and western blot exposure, while β-Tubulin is from the same gel and western blot but at high exposure (see full images in Supplementary information Figure SI-4). (**b**) Cell proliferation measured by MTT assay on HCPEpiCs. Cells were incubated for 2.5 and 5 days with 5 μg/ml of untreated Cp, Cp-ox/de or BSA-ox/de. Cells number was reported as percentages of the seeded cells. Statistical significance p value was evaluated by one way ANOVA test (mean ± SE; n = 3, in triplicates) with post analysis Tukey’s test for comparison of all pairs of groups (**p < 0.01).

The binding of Cp-ox/de to integrins expressed by HCPEpiCs cells is able to trigger an intracellular signalling (Fig. 6a). Western blot analysis of molecules representative of the integrin signalling pathways, showed, upon Cp-ox/de stimulation, an increase in the phosphorylation of the activation residues of p-Tyr^397^FAK1 and p-Thr^202^Tyr^204^ERK1/2, accompanied by the phosphorylation of inhibitory residue p-Ser^9^GSK3β, while no phosphorylation of the Ser^473^Akt residue was observed (Fig. 6a). These results suggest that in HCPEpiCs the signal mediated by Cp-ox/de via integrin engagement is addressed to the regulation of cell cycle, proliferation and MAPK signaling pathway. Indeed, HCPEpiCs treated with Cp-ox/de showed a proliferation reduction/arrest at day 5 (p < 0.0001, one way ANOVA; Tukey's post test analysis at day 5: p < 0.01 for Cp-ox/de vs. Cp; Cp-ox/de vs. BSA-ox/de) (Fig. 6b). The inhibitory effect detectable at longer incubation time, compared to HaCaT, might be due to the low proliferation rate of these primary cells. The proliferation inhibition was not observed when cells were treated either with untreated Cp or control BSA-ox/de (p < 0.0001, one way ANOVA; Tukey's post test analysis for 2.5 and 5 days comparison: p < 0.0001 for Cp and BSA-ox/de; p = not significant for Cp-ox/de) (Fig. 6b). These data confirmed the hypothesis that signalling elicited by the interaction of Cp-ox/de with integrin may modulates proliferation/vitality of epithelial cells also within CNS^15^.

## Discussion

In this study we hypothesized that integrins engagement by Cp-ox/de, via the isoDGR-motifs, transduces a signalling that alters the physiology of the epithelial cells. The hypothesis was based on the analysis of the molecular pathways of activation elicited by the interaction of Cp-ox/de with integrin, that we made by reverse phase protein array approach^15^. The results confirmed that epithelial cells were affected in terms of proliferation and cell cycle arrest, which in turn resulted in apoptosis induction.

The assumption that proliferation inhibitory signalling was elicited by isoDGR/integrin binding was supported by the inhibition switch off induced by PIMT-treatment. This enzyme is able to converts the isoDGR-motif, suitable to bind the RGD binding pocket of integrins by structurally mimicking the RGD-motif, to the DGR-motif, structurally inappropriate for integrin binding^15,17–19^. The observed anti-proliferative effect occurred regardless if the oxidation/deamidation of Cp was induced chemically or by incubation in the oxidative PD-CSF environment, suggesting that Cp modifications may be promoted in the pathological conditions. Indeed, asparagine deamidation is a spontaneous reaction that occurs during protein aging and that can be accelerated by oxidative environment^20,21^. In PD patients, Cp oxidation and deamidation could be fostered by hydrogen peroxide, which has been found at higher concentration in the patient's CSF, compared to the concentration found in the CSF of healthy subjects^16^. The possible role of hydrogen peroxide in fostering deamidation of Cp NGR-motifs is also supported by the evidence that catalase prevented the acquisition of integrin binding properties by purified Cp spiked in pathological CSF^16^. In the CSF of PD patients, endogenous Cp shows about 20% higher rate of deamidation at the ^962^NGR-motif, compared to healthy subjects CSF, which is in turn converted into the isoDGR-motif able to bind integrins^5,15,16^. Therefore, integrins engagement by deamidated NGR-motifs might be a mechanism of Cp function switch that occurs in vivo under pathological conditions which imply oxidative stress. Interestingly, the deamidation of the same ^962^NGR-motif of the Cp has been reported to occur in the serum of type 2 diabetes patients^22^, that is characterized by pro-oxidant pathological environment as well the CSF of neurodegenerative diseases.

Integrins binding can activate different intracellular signalling cascades, which regulate a range of cellular behaviors including cytoskeletal rearrangement, cell polarity, differentiation, growth, survival and apoptosis^23–26^. The Cp-ox/de mediated proliferation inhibition observed in the HaCaT epithelial cell line and primary HCPEpiC was emphasized in HaCaT cells by the cell cycle arrest in the G0/G1 phase and by the consequent induction of apoptosis. Similar detrimental effect of cell cycle arrest in the G1/S transition phase has been reported also by an aberrant signalling mediated via integrin engagement by fibrillar amyloid-β, which leads neuronal death by apoptosis^27,28^. Thus, anomalous/aberrant integrins stimuli occurring under pathological conditions may have negative consequences on epithelial cells proliferation. In epithelial cells, integrins transduce both outside-in and inside-out signalling that regulates the interaction with basal lamina and extracellular matrix (ECM), the organization of focal adhesion and cytoskeletal rearrangement^25^. Alterations of the epithelial cell anchorage to ECM could also be responsible for the observed cell cycle arrest and proliferation inhibition. In fact, cell cycle arrest and apoptosis induction through inappropriate/aberrant integrins engagement is reminiscent of anoikis, the epithelial cells specific apoptosis that depends on the alteration of integrin-mediated anchorage to the extracellular matrix^29–31^. Therefore, is conceivable that inappropriate integrins engagement by isoDGR-motifs of Cp-ox/de might trigger apoptosi signal, as suggested by the inhibition of PI3K/Akt pro-survival signal^26,29,31^ underlined by the Akt phosphorylation inhibition reported in HaCaT cells^15^ and by the lack of Akt phosphorylation observed in this work in HCPEpiC.

Deamidation of NGR motifs present in the ECM proteins has been reported in human pro-oxidant pathological environments of the atherosclerotic plaques, where mediate enhanced monocyte adhesion via αVβ3 integrin binding^32^ which may contribute to plaques progression. This observation supports the hypothesis that aberrant/anomalous integrins engagement by deamidated NGR-motifs of the ECM might be a mechanism of protein function's switch that occurs in vivo under pathological conditions, which imply oxidative stress.

Within the central nervous system, the potential targets of deamidated Cp found in the CSF of neurodegenerative diseases include the epithelial cells of the ependymal layer and of the choroid plexus, which are directly in contact with the CSF. This hypothesis is supported, at least for the CPEpiCs, by the evidences that the interaction with Cp-ox/de alters their functions inducing a cell proliferation inhibition.

At the level of choroid plexus, the CPEpiCs monolayer is important because constitute the so call blood-CSF-barrier (BCSFB). CPEpiCs determine the composition of CSF by the secretion of proteins and metabolites, and by regulating the BCSFB permeability through tight-junctions^33,34^. The histological organization and barrier permeability functions of CPEpiCs have been reported to be altered in aging and in neurodegenerative diseases^35–38^. Thus, the alteration of CPEpiCs physiology we found can lead to BCSFB dysfunction and changes in the CSF composition that might contribute to the pathological mechanisms. Further investigations are needed to assess whether the interaction of Cp-ox/de with CPEpiCs, affecting their functions may also alter BCSFB properties.

In conclusion, in this study we report that Cp-ox/de, generated in the oxidative environment of the CSF from PD patients, affects epithelial cells physiology in terms of cell proliferation, cell cycle arrest and apoptosis induction. These effects presumably depend on the interaction of the Cp isoDGR motifs with the integrins expressed on epithelial cells. Therefore, Cp modifications induced by pathological oxidative environment might contribute to the mechanisms of neurodegeneration via functional gain of integrin binding properties that enables aberrant/inappropriate intracellular signalling in epithelial cells, which in turn induces apoptosis and might represent a novel mechanism with potentially important pathological implications.

## Methods

### CSF samples

CSF samples of PD patients (PD-CSF) and healthy control subjects (H-CSF) were obtained from the Institute of Experimental Neurology Biobank (IRCCS-San Raffaele Hospital)^16^. All the research was carried out in accordance with relevant guidelines and regulations. The experimental protocol was approved by the San Raffaele Hospital—Ethical Committee (Protocol, GO/URC/ER/mm prot. N. 851/DG, 06/10/2009 version). Informed consent was obtained from all patients or their Legal guardians if they were unable, and from the healthy control subjects.

Samples were collected by lumbar puncture, centrifuged to eliminate cells, and stored at − 80 °C in an N_2_-supplemented atmosphere to avoid oxidation. Demographic/clinical features of patients and control subjects are reported in^16^.

### Cell cultures and reagents

HaCaT cells, a human keratinocyte epithelial cell line (America Type Culture Collection) were cultured under standard conditions as reported^15^. The primary human choroid plexus epithelial cells (HCPEpiCs) (ScienCell Research Laboratories) were cultured in EpicM epithelial cell medium added with 1% epithelial cell growth supplement (ScienCell Research Laboratories) and 2% fetal bovine serum (FBS). HCPEpiCs cells were used within three cell-culture passages to exclude de-differentiation to mesenchymal cells.

Chemical reagents were from Sigma, if not specified. Human plasma purified Cp was from Enzo Life Sciences. The antibodies used were: anti-ceruloplasmin (ab8813, Abcam); the antibodies used for the signaling analysis (FAK #3285, p-Tyr^397^FAK #3283, ERK1/2 #9102, p-Thr^202/^Tyr^204^ERK1/2 #9101, GSK3β #9315, p-Ser^9^GSK3β #9336, AKT #4691, p-Ser^473^AKT #193H12) were from Cell Signaling Technology, while anti-β-tubulin antibody (T6199) was from Sigma-Aldrich.

### Ceruloplasmin oxidation and aging treatments

Accelerated aging under oxidative conditions of Cp and bovine serum albumin (BSA) was obtained incubating both proteins for 16 h at 37 °C in 100 mM ammonium bicarbonate buffer, pH 8.5, containing 10 mM H_2_O_2_. This treatment is known to favour asparagine deamidation of NGR sites of Cp^15^. Both products were then dialyzed against PBS and the resulting products, referred in the text as oxidized/deamidated-Cp (Cp-ox/de) and BSA (BSA-ox/de), respectively, were then stored at − 80 °C for further experiments. Aging of purified Cp in CSF was carried out by adding an aliquot of Cp (20 μg/ml, final concentration) to a pool of CSF from either healthy subjects or PD patients following incubation at 37 °C for 9 days, under N_2_-conditioned atmosphere to avoid the exposure to oxidative environment. Cp exposed to CSF was then immunoprecipitated using protein-G Agarose Beads (Invitrogen) loaded with anti-Cp antibody (ab8813, abcam), chemically cross-linked to the beads with 20 mM dimethyl pimelimidate. After 12 h stirring incubation at 4 °C, beads were washed with PBS and bound Cp was eluted with 0.1 M glycine, pH 2.5. Eluted samples were immediately diafiltered with PBS using an Amicon filter device (30 KDa cut-off) and protein concentration was quantified by Bradford assay. For the referred "0 day" of incubation, Cp was added to the CSF samples and straight after purified as described above.

### Cell proliferation assay, cell cycle analysis and apoptosis analysis

HaCaT and HCPEpiC cell proliferation assay was carried out as follow: cells were seed in 96-well plates (100 μl, 10,000 cells/well) in medium containing 0.1% FBS and culture for 24 h in 5% CO_2_ at 37 °C for starvation. Then, depending on the type of assay, cells were cultured for different times (24–72 h, HaCaT; 60–120 h HCPEpiC) in the presence of various amount of Cp, Cp-ox/de, or BSA-ox/de (0, 1, 3, 5, 7, 20 μg/ml). In parallel, HaCaT cells were also cultured for 24–72 h in the presence of Cp (2 μg/ml) immunoprecipitated after aging in H-CSF or PD-CSF. At the end of the incubation time, cell proliferation/viability was determined by adding 10 μl of 3-(4,5-dimethylthiazol-2-yl)-2,5-diphenyltetrazolium bromide (MTT)^39^ solution (5 mg/ml in PBS) to each well. After 2 h of incubation at 37 °C under 5% CO_2_, the supernatant was removed using a pasteur pipette connected to a vacuum pump, and 200 μl of dimethyl sulphoxide were added to each well to dissolve the formazan crystals. The absorbance at 570 nm was then measured using a plate reader. Quantification of cell number was determined using an external standard curve prepared by plating various amount of HaCaT cells (range 0–10^5^ cell/well, in quadruplicate, 12 serial dilution 1:2) in a 96 well plate on the day of the assay. The cell viability data are reported as percentage of seeded cells.

For PIMT treatment, Cp-ox/de (20 μg/ml) was incubated at 37 °C for 16 h in *assay buffer* consisting of 50 mM sodium phosphate buffer, pH 6.8, containing 0.02 mM *S*-adenosyl-l-methionine and 5 μl of PIMT enzyme solution (IsoQuant isoaspartate detection kit; Promega). PIMT-treated proteins were used in proliferation assays at 5 μg/ml. In parallel, *assay buffer* alone (i.e. lacking the Cp-ox/de) was used as negative control.

Cell cycle and cell apoptosis quantification were carried out using HaCaT cells incubated with the different proteins (5 μg/ml). Cells were labelled with a thymidine analog to separate cells in S phase from those in G0/G1 and G2/M exploiting the Click-iT EdU Flow Cytometry AssayKit, and with the Vybrant DyeCycle Violet dye (Invitrogen) for total DNA detection. Apoptosis was evaluated by Annexin V FITC kit staining (Immunological Sciences). Labelled cells were then analyzed by flow cytometry using an LSR Fortessa cell cytofluorimeter (Becton Dickinson).

### HCPEpiC adhesion assay and PIMT treatment

96-well polyvinyl chloride microtiter plates were coated with untreated Cp or Cp-ox/de (2, 5, 20 μg/ml in PBS, 16 h at 4 °C). After washing and blocking (3% BSA in PBS), plates were seeded with HCPEpiCs (40,000 cells/well in 0.1% BSA DMEM), and left to adhere (3 h at 37 °C). Adherent cells were fixed and stained with crystal violet, and adhesion evaluated by absorbance at 570 nm^15^. For PIMT treatment, plates coated as described above were washed and filled with 45 μl of 0.02 mM *S*-adenosyl-l-methionine in 50 mM Na_3_PO_4_, pH 6.8, and 5 μl of PIMT solution (IsoQuant isoaspartate detection kit; Promega), and incubated at 37 °C for 16 h. Then plates were washed and cell adhesion assay performed as described above.

### Western blot analysis

For signaling analysis, HCPEpiCs cells in culture were incubated 2 h with 5 μg/ml of either untreated Cp or Cp-ox/de and solubilized in lysis buffer (50 mM TRIS, 150 mM NaCl, 1 mM EDTA, 1% Triton-X, 0,1% SDS, protease inhibitors cocktail, and 25 mM NaF and 5 mM NaVO_3_ as phosphatase inhibitors) and quantified with Bradford assay (Bio-Rad). Subsequently, cell lysates were resolved by 10%-acrylamide SDS-PAGE and analyzed by Western blot using specific antibodies (see above) whose reactivity was detected by using appropriate HRP-conjugated secondary antibodies followed by enhanced chemiluminescence reaction (ECL reagent, GE Healthcare) and film exposure.

### Statistical analysis

All statistical analyses were carried out with Prism software V4.03 (GraphPad). We evaluated the statistical significance among three or more groups by One-way Analysis of Variance (ANOVA), if the data passed the normality test for Gaussian distribution (Kolmogorov–Smirnov test), or by Kruskal–Wallis test. Ad hoc post-test analysis was performed for comparison of all pairs of groups or for comparison of selected pairs of groups, the Tukey’s or Bonferroni's test, respectively, were used for the ANOVA, while the Dunn’s test was used in the case of Kruskal–Wallis analysis. In all analyses a p value < 0.05 was considered as statistically significant.

The statistical significance of two-tailed p value for the comparison of two means and standard error, was evaluated by Mann–Whitney test. In all analyses a p value < 0.05 was considered statistically significant.

## Supplementary information

Supplementary Information 1.

## Data Availability

The datasets used and/or analysed during the current study are available from the corresponding author on reasonable request.
