# Supplementary material for: Ceruloplasmin oxidized and deamidated by Parkinson's disease cerebrospinal fluid induces epithelial cells proliferation arrest and apoptosis
Source: Sci Rep. 2020 Sep 23;10:15507. doi: 10.1038/s41598-020-72447-z (PMC7511351; doi:10.1038/s41598-020-72447-z)
Supplement: Supplementary file 1 — Supplementary Information 1. [file 41598_2020_72447_MOESM1_ESM.pdf]

Supplementary Information

Ceruloplasmin oxidized and deamidated by Parkinson's disease cerebrospinal fluid induces epithelial cells proliferation arrest and apoptosis

Marco Barbariga[1], Alan Zanardi[1], Flavio Curnis[2], Antonio Conti[1], Daniela Boselli[3], Simona Di Terlizzi[3], Massimo Alessio[1]

[1]Proteome Biochemistry, [2]Tumor Biology and Vascular Targeting, [3]FRACTAL (Flow cytometry Resource, Advanced Cytometry Technical Applications Laboratory), IRCCS-Ospedale San Raffaele, 20132, Milan, Italy

Supplementary Information Figure SI-1a,b,c

Effect of oxidized/deamidated-Ceruloplasmin (Cp-ox/de) on the HaCaT epithelial cell proliferation measured by *in vivo* time-lapse imaging.

As proof of concept that the results obtained in our study with the MTT assay were not suffering of sensitivity drawbacks, we repeated the proliferation assay using the *in vivo* time-lapse imaging proliferation measurement performed with IncuCyte system (Sartorius). The IncuCyte system records in real-time, within cell-culture incubator, the images of the cells growing in the different wells along the entire proliferation time, and, at specific time points, the area covered by the cells within each optical field (5 for each well for a 96 wells plate) is normalized by the software to the area covered by the cells in the same optical field at time zero.

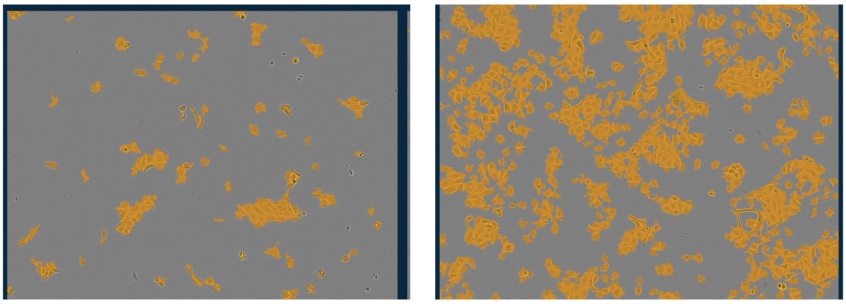

Figure SI-1a. Cell growth as detected by IncuCyte system in the same optical field and in the same well at time 0 *vs.* 48 hours.

We performed the experiment in 6 replicate wells for each condition that were identical as for the figure 1 of the manuscript. The results (see graph below) showed profile of proliferations comparable to the profiles obtained with MTT reported in figure 1a of the manuscript. The profiles, show a clear proliferation inhibition starting at 24 hours only for the cells treated with Cp-ox/de, and the inhibition resulted to be significant at 48 hours in comparison to the others experimental conditions (ANOVA $p<0.0001$; Bonferroni's post test: $p<0.0001$ for Cp-ox/de *vs.* untreated and Cp-ox/de *vs*. Cp; $p<0.001$ for Cp-ox/de *vs.* BSA-ox/de).

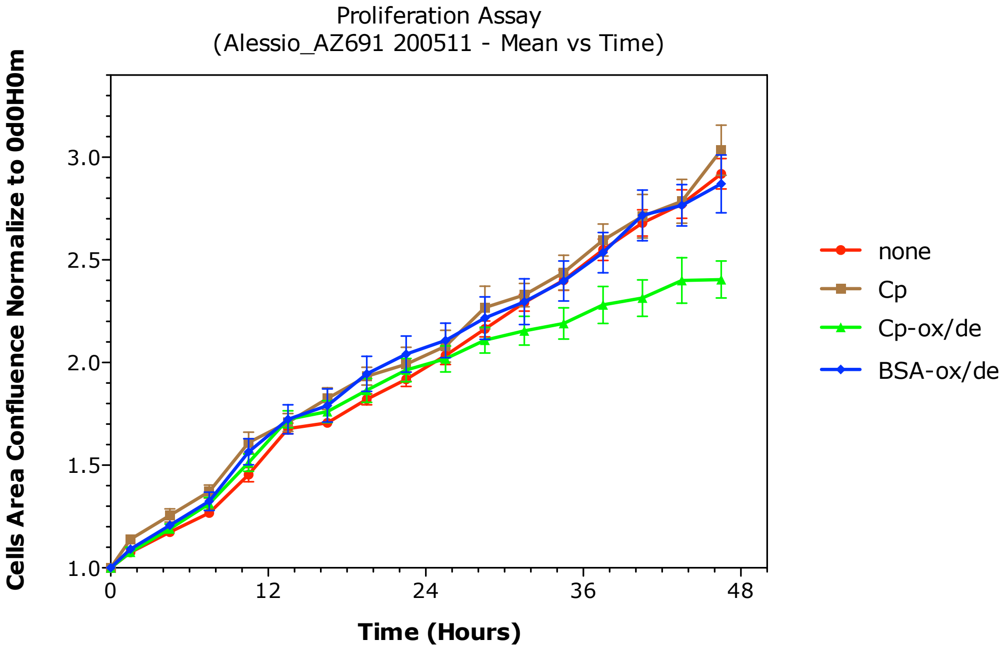

Figure SI-1b. HaCaT cells were incubated for 48 h with 5 μg/ml of Cp-ox/de, untreated Cp, oxidized/deamidated-BSA (BSA-ox/de) or without stimuli (none), and proliferation evaluated by imaging every 3 hours. Proliferation is reported as area covered by the cells at time x normalized by to the area covered by the cells at time zero.

We also evaluated the inhibition of cell proliferation at 48 hour using different concentration of the stimuli 1, 5, 20 μg/ml. Also in this case the dose dependent inhibition profiles obtained for Cp-ox/de with *in vivo* time-lapse imaging is comparable to that one obtained with the MTT assay (see graph below compared to the graph of figure 1b of the manuscript).

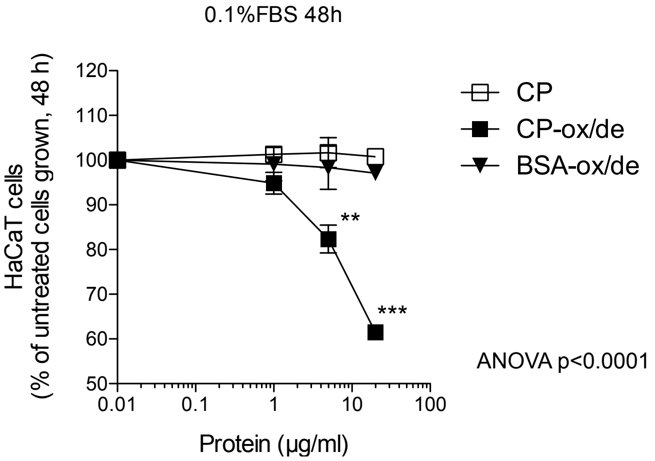

Figure SI-1c. HaCaT cells were incubated for 48 h with 1, 5, 20 μg/ml of the indicated stimuli, or without stimulus. Cell proliferation was determined by *in vivo* time-lapse imaging and reported as percentages of the untreated cells grown at 48 h.

These results confirmed the specific HaCaT cell proliferation inhibition induced by Cp-ox/de.

Supplementary Information Figure SI-2

<u>Analysis of primary human choroid plexus epithelial cells (HCPEpiC) for integrin expression.</u>

Semi-confluent HCPEpiC cells were mechanically detached by scraping and immunostained using the following mouse anti-integrin antibodies: anti-αV subunit (clone P3G8, Millipore), anti-αVβ3 (clone LM609, Millipore), anti-αVβ5 (clone P1F6, Millipore), anti-αVβ6 (clone 10D5, Millipore) and anti-α5 subunit (clone P1D6, Millipore) or an irrelevant isotype-matched control antibody (MOPC-31, Sigma). The binding was detected using a goat anti-mouse Alexa Fluor 488-labeled polyclonal antibody. Sample acquisition and analysis were performed with BD-FACSCanto instrument (BD Biosciences) and FlowJo software (LLC), respectively.

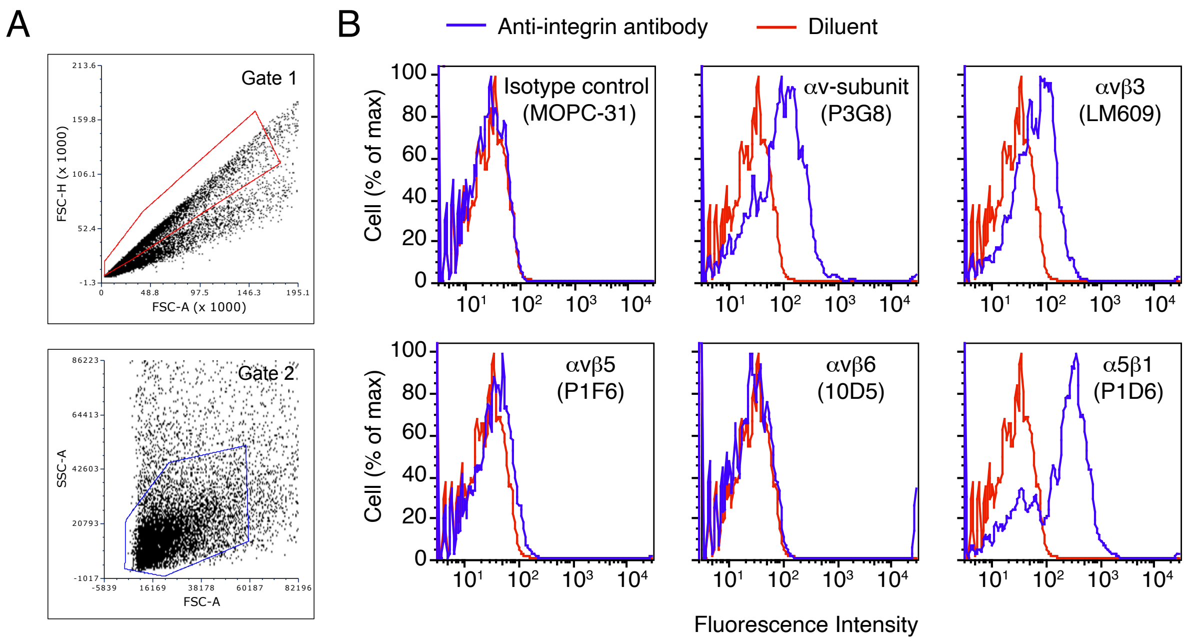

Figure SI-2. Expression of αv-, α5- subunit, αvβ3, αvβ5, and αvβ6 by human choroid plexus epithelial (HCPEpiC), as evaluated by a FACS analysis using the indicated mouse monoclonal antibody, followed by AlexaFluor 488-goat anti-mouse IgG polyclonal antibody. **A**) Dot plots and gating strategy used to characterize the expression of integrins by HCPEpiC cells. *Gate 1* shows single cells selected within the entire cell population based on FSCH (forward scatter high) and FSCA (forward scatter area) parameters. *Gate 2* shows the cells population in *Gate 1* used to characterize the antibody binding reported in panel **B**, based on FSCA versus SSCA parameters.

Supplementary Information Figure SI-3

Gating strategy used to characterized HaCat cells stained with annexin V-FITC conjugate.

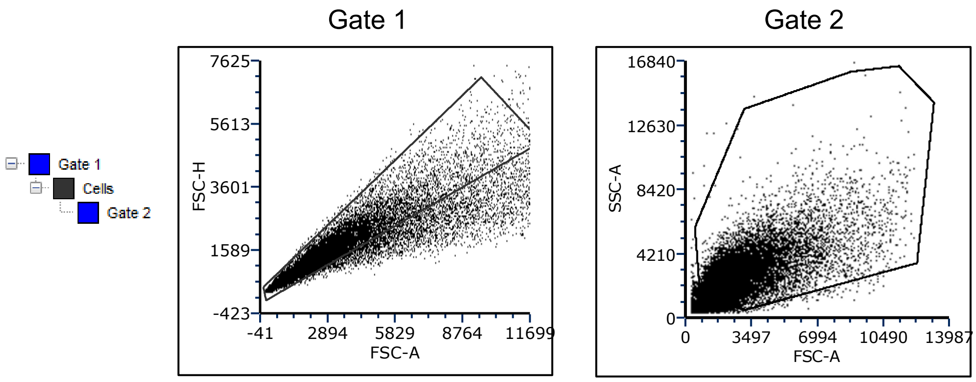

Figure SI-3. Dot plots and gating strategy used to characterize HaCat cells stained with annexin V-FITC conjugate. *Gate 1* shows single cells selected within the entire cell population based on FSCH (forward scatter high) and FSCA (forward scatter area) parameters. *Gate 2* shows the cells population in *Gate 1* used to quantify annexin V-positive cells, based on FSCA versus SSCA parameters.

Supplementary Information Figure SI-4

<u>Full gel/Western blot images that have been used for the generation of cropped panels in the figure 6 of the manuscript.</u>

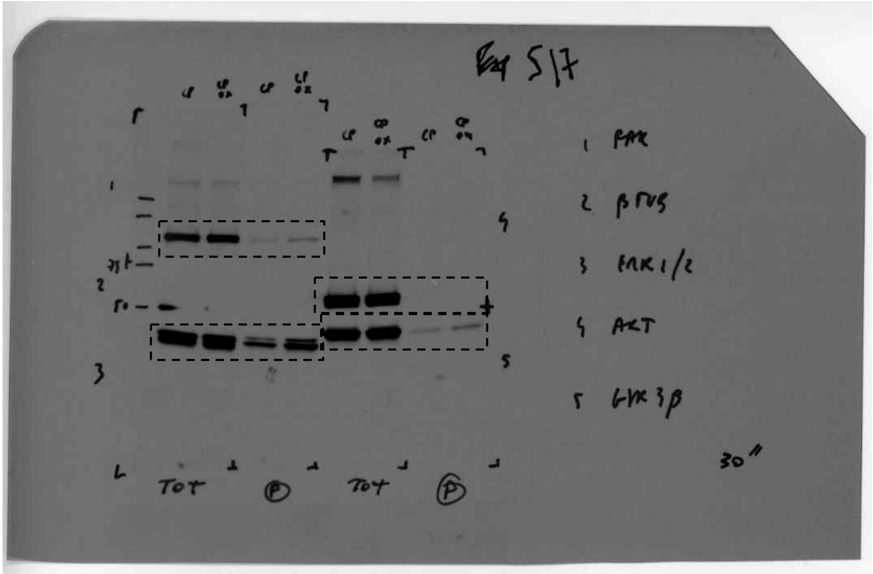

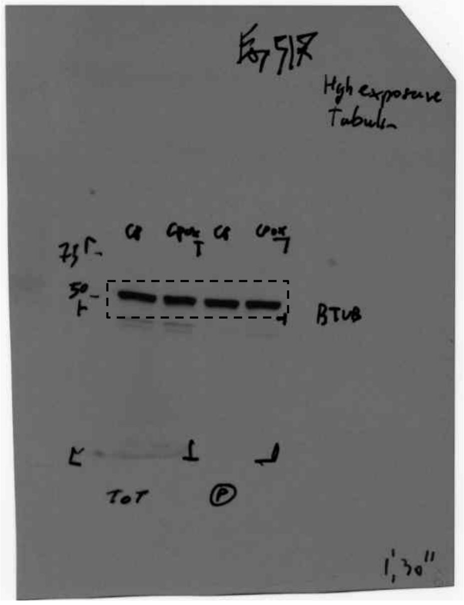

Figure SI-4. Cell lysate from HCPEpiC cells treated with Cp or Cp-de/ox were run on the same SDS-PAGE in multiple lanes with alternate loading (Cp, Cpox). Nitrocellulose was cut for the incubation with different antibodies according to the expected molecular weight for the evaluation of the total protein expression level (ToT) and for the phosphorylation level (P). Nitrocellulose were then recomposed according to the molecular weight for chemiluminescence reaction and film exposure. Bottom panel is the high exposure film used for the β-Tubulin expression. Dashed rectangle identify the cropped areas used for the generation of figure 6 of the manuscript.
